# Usefulness of intravoxel incoherent motion MRI for visualizing slow cerebrospinal fluid motion

**DOI:** 10.1186/s12987-023-00415-6

**Published:** 2023-03-10

**Authors:** Shigeki Yamada, Shinnosuke Hiratsuka, Tomohiro Otani, Satoshi Ii, Shigeo Wada, Marie Oshima, Kazuhiko Nozaki, Yoshiyuki Watanabe

**Affiliations:** 1 Department of Neurosurgery, Nagoya City University Graduate School of Medical Science, 1 Kawasumi, Mizuho-Cho, Mizuho-Ku, Nagoya, Aichi 467-8601 Japan; 2 Department of Neurosurgery, Shiga University of Medical Science, Shiga, Japan; 3 Normal Pressure Hydrocephalus Center, Rakuwakai Otowa Hospital, Kyoto, Japan; 4 Interfaculty Initiative in Information Studies/Institute of Industrial Science, The University of Tokyo, Tokyo, Japan; 5 Department of Radiology, Shiga University of Medical Science, Shiga, Japan; 6 Department of Mechanical Science and Bioengineering, Graduate School of Engineering Science, Osaka University, Osaka, Japan; 7 Faculty of System Design, Tokyo Metropolitan University, Tokyo, Japan

**Keywords:** Cerebrospinal fluid dynamics, Idiopathic normal pressure hydrocephalus, Aging, Intravoxel incoherent motion, Diffusion-weighted image

## Abstract

**Background:**

In the cerebrospinal fluid (CSF) dynamics, the pulsations of cerebral arteries and brain is considered the main driving force for the reciprocating bidirectional CSF movements. However, measuring these complex CSF movements on conventional flow-related MRI methods is difficult. We tried to visualize and quantify the CSF motion by using intravoxel incoherent motion (IVIM) MRI with low multi-b diffusion-weighted imaging.

**Methods:**

Diffusion-weighted sequence with six b values (0, 50, 100, 250, 500, and 1000 s/mm^2^) was performed on 132 healthy volunteers aged ≥ 20 years and 36 patients with idiopathic normal pressure hydrocephalus (iNPH). The healthy volunteers were divided into three age groups (< 40, 40 to < 60, and ≥ 60 years). In the IVIM analysis, the bi-exponential IVIM fitting method using the Levenberg–Marquardt algorithm was adapted. The average, maximum, and minimum values of ADC, D, D*, and fraction of incoherent perfusion (*f*) calculated by IVIM were quantitatively measured in 45 regions of interests in the whole ventricles and subarachnoid spaces.

**Results:**

Compared with healthy controls aged ≥ 60 years, the iNPH group had significantly lower mean f values in all the parts of the lateral and 3rd ventricles, whereas significantly higher mean f value in the bilateral foramina of Luschka. In the bilateral Sylvian fossa, which contain the middle cerebral bifurcation, the mean f values increased gradually with increasing age, whereas those were significantly lower in the iNPH group. In the 45 regions of interests, the f values in the bilateral foramina of Luschka were the most positively correlated with the ventricular size and indices specific to iNPH, whereas that in the anterior part of the 3rd ventricle was the most negatively correlated with the ventricular size and indices specific to iNPH. Other parameters of ADC, D, and D* were not significantly different between the two groups in any locations.

**Conclusions:**

The *f* value on IVIM MRI is useful for evaluating small pulsatile complex motion of CSF throughout the intracranial CSF spaces. Patients with iNPH had significantly lower mean *f* values in the whole lateral ventricles and 3rd ventricles and significantly higher mean f value in the bilateral foramina of Luschka, compared with healthy controls aged ≥ 60 years.

## Background

The cerebrospinal fluid (CSF) motion is composed of a steady microflow produced by the rhythmic wavy movement of motile cilia on the ventricular wall surface [1, 2], a dynamic pulsatile flow produced by the pulsations of cerebral arteries and brain [2–7], and an uncertain flow produced by respiration and head movement [8–11]. This complex CSF motion was considered to decrease and stagnate with aging because of declines in brain volume, arterial elasticity, and circulating cerebral blood volume [12]. Age-related declines in CSF metabolism impair the excretion of neurotoxic waste products from the brain and were associated with dementia and neurodegenerative disorders [13]. In the glymphatic system [14–16], CSF flows from the subarachnoid spaces into the brain through periarterial spaces surrounding the penetrating cerebral arteries, exchanges with interstitial fluid, flows from the periarterial spaces to the perivenous spaces, re-exchanges with CSF, and drains from the perivenous space to the subarachnoid spaces or ventricles. This complex pulsatile motion of the CSF and interstitial fluid is mainly driven by arterial pulsations [13, 17]. However, visualizing and quantifying the small bidirectional pulsatile CSF motion with a velocity of < 1 cm/s had been difficult by using recent flow-related MRI methods, such as conventional phase-contrast MRI [3, 4, 18], four-dimensional flow MRI [5, 6], and time-spatial labeling inversion pulse (time-SLIP) MRI [8–10]. In these previous CSF dynamic studies on MRI, the CSF motion was observed only in the cerebral aqueduct, foramina of Monro, prepontine cistern, and craniocervical junction, but not in the convexity part of the subarachnoid spaces. Thus, CSF was thought to be not flowing in the convexity part of the subarachnoid space. In the glymphatic system [14–16], however, CSF in the convexity part of the subarachnoid space flows rapidly into the deep parenchyma along the paravascular routes, although that has not been fully demonstrated in the CSF dynamics study on MRI.

A diffusion weighted image (DWI) with a lower b value could detect CSF motion as a signal attenuation or dephasing [19]. Taoka et al. reported that a b value of 500 s/mm^2^ in DWI was useful for evaluating CSF motion in the whole cranium [20, 21]. They also proposed a new method that combined DWIs with multiple b values of 0, 50, 100, 200, 300, 500, 700, and 1000 s/mm^2^, which applies the phenomenon that the motion-related signal dephasing of the CSF in a wider area with small-to-large CSF motion increases as the b value in DWI increases from 0 s/mm^2^ [22]. As an imaging method for the quantification of diffusion and perfusion (nonuniform microflow) by using multiple low b values of DWIs, intravoxel incoherent motion (IVIM) MRI developed by Le Bihan et al. [19, 23] has been applied in clinical practice, especially in the field of oncology [23–28]. Incoherent motion is defined as diffusion, i.e., microscopic random translational motion of fluid molecules, whereas the multidirectional complex, laminar, and turbulent flows in CSF spaces are included in coherent motion. Based on the evidence of these reported studies, we considered that IVIM MRI could be applied to the complex small CSF motion. This study aimed to investigate whether the complex CSF motion in all parts of the ventricles and subarachnoid spaces including the convexity sulci could be evaluated quantitatively by IVIM MRI and to compare them between patients with idiopathic normal pressure hydrocephalus (iNPH) and healthy volunteers. The second objective is to prove the association between IVIM parameters and CSF distribution in iNPH and healthy aging brain.

## Materials and methods

### Study population

The study design and protocol have been approved by the ethics committees for human research at our institute (IRB number: R2019-227). This study followed a prospective and observational design. The study was performed in accordance with the approved guidelines of the Declaration of Helsinki. From November 2020 to February 2022, 133 healthy volunteers aged ≥ 20 years underwent MRI after providing written informed consent explaining the potential for detection of brain disease. Volunteers were recruited from medical staff and students, and their families by open recruitment. Inclusion criteria for this study were those who had no history of brain injury, brain tumor or cerebrovascular disease on previous brain MRI, or those who had never undergone brain MRI and no neurological symptoms including cognitive function. One volunteer aged 84 years old was excluded from this study because of a history of head surgery due to a head injury over 30 years ago. In addition, three volunteers were incidentally found small unruptured intracranial aneurysms with a maximum diameter of < 2 mm on this MRI. They were included in this study, because small unruptured aneurysms might not affect CSF motion.

Patients’ MRI data was used in an opt-out method, after their personal information was anonymized in a linkable manner. Among 44 patients suspected with NPH, 5 patients diagnosed with secondary NPH [29] that developed after subarachnoid hemorrhage [3], intracerebral hemorrhage [1], and severe meningitis [1], and 3 patients diagnosed with congenital/developmental etiology NPH [30] were excluded from this study. Finally, 36 patients diagnosed with iNPH who had radiological findings of disproportionately enlarged subarachnoid space hydrocephalus (DESH) [31], specifically ventricular dilatation, enlarged Sylvian fissure, and narrow sulci at the high convexity, and triad symptoms of gait disturbance, cognitive impairment, and/or urinary incontinence were included in this study, according to the Japanese guidelines for management of iNPH [32]. Of them, 18 patients (50%) underwent CSF removal in 30–35 ml via a lumbar tap and were evaluated for changes in their symptoms before, one day and two days after the CSF tap test. In addition, 21 patients (86%) underwent CSF shunt surgery and their symptoms improved by ≥ 1 point on the modified Rankin Scale and/or the Japanese iNPH grading scale [32].

### Image acquisitions

All MRI examinations were performed using a 3-Tesla MRI system (Discovery MR 750W, GE Medical Systems, Inc. Chicago, IL, USA). DWI was performed in axial planes by using six b values (0, 50, 100, 250, 500, and 1000 s/mm^2^). The DWI sequence parameters were as follows: repetition time, 6666 ms; echo time, 78 ms; flip angle, 90°; bandwidth, 1305 Hz/Px; slice thickness, 3.0 mm; field of view, 220 mm; acquisition matrix size, 128 × 192; pixel size, 1.7 × 1.1 mm; acquisition time, around 2 min for total acquisition; motion-probing gradient, bipolar type. The isotropic images were created from 3 orthogonal diffuse gradient pulse images. The sequence parameters for 3D T2-weighted fast spin-echo Cube sequence were as follows: TR, 2,000 ms; TE, 85.3 ms; matrix 288 × 288; voxel size, 0.8 × 0.8 × 0.8 mm; and acquisition time, approximately 4 min.

### Data processing

**Fig. 1:**
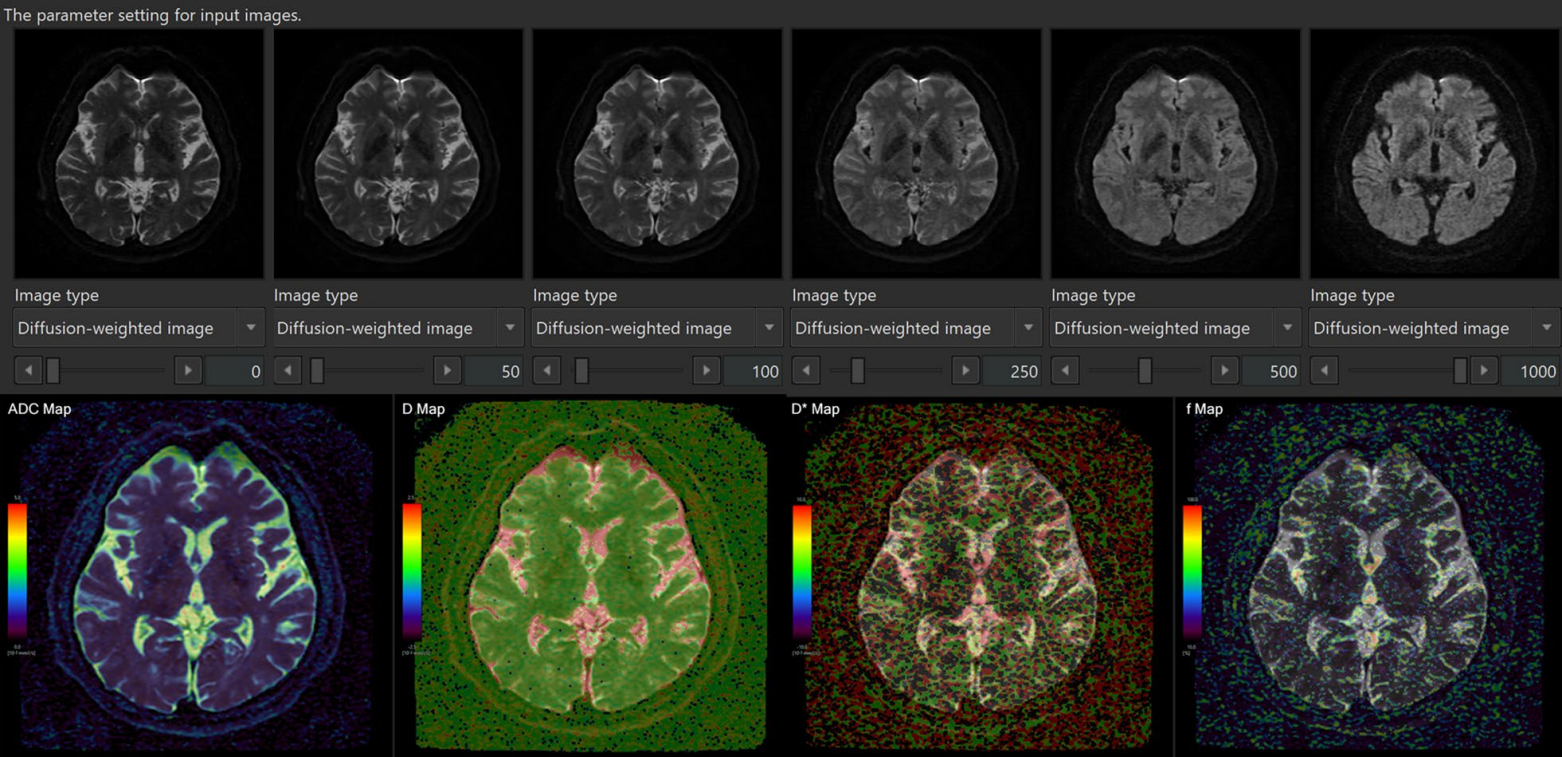
Schema of our methods of intravoxel incoherent motion MRI

IVIM analysis was performed using the IVIM application on an independent 3D volume analyzer workstation (SYNAPSE 3D; FUJIFILM Corporation, Tokyo, Japan). Four maps of apparent diffusion coefficient (ADC, mm^2^/s), true diffusion coefficient (D, mm^2^/s), pseudo-diffusion coefficient (D*, mm^2^/s), and fraction of incoherent perfusion (*f*, %) in one voxel were automatically created approximately 30 s after reading the multi-b DWI sequence on the IVIM application, as shown in Fig. 1.

Signal decay was estimated by using the following bi-exponential equation:

$$\frac{S}{S0}=f\cdot \mathrm{exp}\left(-b{D}^{*}\right)+\left(1-f\right)\cdot \mathrm{exp}\left(-bD\right)$$

**Fig. 2:**
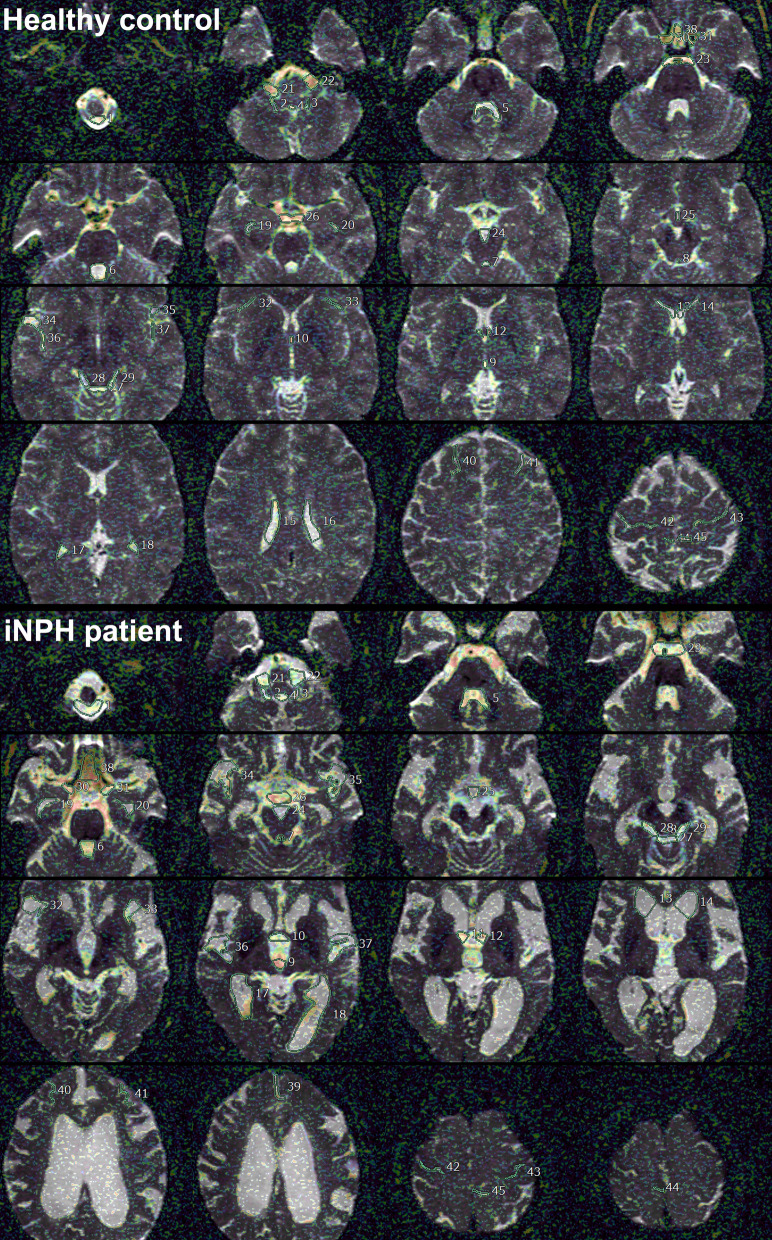
Location of the 45 regions of interest on* f* map (%) of IVIM MRI in the ventricles and subarachnoid spaces in a representative healthy control (top) and iNPH patient (bottom)

Here, *S* is the signal intensity at a given b value, *S*0 is the signal intensity at b = 0 s/mm^2^, and ADC was calculated by nonlinear least-squares minimization. The bi-exponential IVIM fitting method using the Levenberg–Marquardt algorithm was performed with the constraint on *f* (0 < *f* < 1). In the normal brain, *f* has been reported to be generally less than 5% and D is approximately 0.002 mm^2^/s [23, 25]. After IVIM analysis, b = 0 DWI images were automatically superimposed on ADC, D, D* and *f* maps (Fig. 1 lower column). Because the shape of the ventricles and the subarachnoid spaces in the iNPH and control groups had laterality and individual differences, the 45 regions of interests (ROIs) in the whole ventricles and subarachnoid spaces were manually determined by a single researcher (S.Y.) based on anatomical features as shown in Fig. 2 and Table 2.

All of the participants were measured indices specific to iNPH on the 3D T2-weighted fast spin-echo MRI. The Evans index was measured as the maximal width of the frontal horns of the lateral ventricles to the maximal width of the internal diameter of the cranium based on the x-dimension, and the Z-Evans index was measured as the maximum z-axial length of the frontal horns of the lateral ventricles to the maximum cranial z-axial length on the coronal plane, which was perpendicular to the anteroposterior commissure plane on the anterior commissure [33].

The brain per ventricle ratios (BVRs) were measured as the maximum width of the brain just above the lateral ventricles divided by the maximum width of the lateral ventricles on the reference coronal planes at the anterior commissure and posterior commissure levels, respectively [34]. The callosal angle was measured as the angle of the roof of the bilateral ventricles on the coronal plane at the posterior commissure level [35]. In addition, the total ventricles and subarachnoid spaces were segmented from the 3D T2-weighted Cube sequence in our original method combined a simple threshold algorithm and manual segmentation, as previously reported [29, 33, 34].

The average, maximum, and minimum values of ADC, D, D*, and *f* were automatically measured in each ROI. The numbers indicate the following regions: the foramen magnum [1], bilateral foramina of Luschka (right 2 and left 3), foramen of Magendie [4], 4th ventricle (lower 5, middle 6, and upper 7), cerebral aqueduct [8], 3rd ventricle (anterior 9 and posterior 10), bilateral foramina of Monro (11 and 12), bilateral lateral ventricles (anterior horn 13 and 14, body 15 and 16, trigone 17 and 18, and inferior horn 19 and 20), bilateral cerebellopontine angle (21 and 22), prepontine cistern (23), interpeduncular cistern (24), lamina terminalis cistern (25), suprasellar or chiasmatic cistern (26), quadrigeminal cistern (27), bilateral ambient cisterns (28 and 29), bilateral carotid cisterns (30 and 31), bilateral Sylvian sulci (anterior 32 and 33, and posterior 36 and 37), bilateral Sylvian fossae (34 and 35), interhemispheric fissure (lower 38 and upper 39), bilateral superior frontal sulci (40 and 41), bilateral central sulci (42 and 43), and bilateral marginal sulci (44 and 45).

### Statistical analysis

Mean values ± standard deviations (SD) for age and several measurements in patients with iNPH were compared with those in healthy controls using the Mann–Whitney–Wilcoxon test. Fisher’s exact test was used to compare the proportions of the two groups. To investigate the trends in mean *f* values due to aging in healthy controls, mean values ± SD for several measurements among three age groups (< 40, 40 to < 60, and ≥ 60 years) were compared using the Kruskal–Wallis test. Significance was assumed at a probability (*P*) value of < 0.001. All missing data points were treated as deficit data that did not affect other variables. Statistical analyses were performed using the R software version 4.1.0 (R Foundation for Statistical Computing; http://www.R-project.org).

## Results

In total, the study included 168 participants, comprising 36 patients diagnosed with iNPH (mean age, 76.8 ± 7.5 years; range, 61–88 years; 25 males, 11 females) and 132 healthy volunteers (mean age, 47.5 ± 16.9 years; range, 21–92 years; 46 males, 86 females) (Table 1).

**Table 1:** Clinical characteristics of the study population

	Healthy volunteer			iNPH patient	*P*-value^a^	*P*-value^b^
	< 40 years	40–59 years	≥ 60 years			
Total number	47	49	36	36		
Sex (male: female)	16:31	15:34	15:21	25:11	0.032	0.577
Mean age (years)	29.3 ± 5.5	49.1 ± 5.9	69.3 ± 6.8	76.8 ± 7.5	< 0.001	< 0.001
Total subarachnoid space (mL)	213.7 ± 44.3	244.1 ± 52.4	305.6 ± 58.0	291.2 ± 61.4	0.336	< 0.001
Total ventricle (mL)	21.3 ± 7.1	23.7 ± 8.3	37.7 ± 14.2	130.1 ± 38.8	< 0.001	< 0.001
Evans index	0.25 ± 0.02	0.25 ± 0.02	0.26 ± 0.02	0.37 ± 0.20	< 0.001	0.034
Z-Evans index	0.24 ± 0.03	0.24 ± 0.03	0.27 ± 0.04	0.41 ± 0.04	< 0.001	< 0.001
BVR at AC	2.1 ± 0.3	2.1 ± 0.3	1.8 ± 0.4	0.8 ± 0.1	< 0.001	< 0.001
BVR at PC	4.8 ± 1.4	4.3 ± 1.1	3.0 ± 1.0	1.0 ± 0.2	< 0.001	< 0.001
Callosal angle (degree)	119.9 ± 9.2	118.6 ± 11.3	117.1 ± 11.5	68.1 ± 17.3	< 0.001	0.594

Gender differences were compared by Fisher's exact test

*AC* anterior commissure, *PC* posterior commissure

^a^*P*-value: probability value for the Mann–Whitney-Wilcoxon test between iNPH patients and healthy volunteer aged ≥ 60 years

^b^*P*-value: probability value for the Kruskal–Wallis test among 3 age groups of healthy volunteer

In the healthy volunteers aged < 60 years, the mean volume of total ventricle was < 25 mL, which was about 10% of the volume of total subarachnoid space, and it increased significantly to 38 mL in the those aged ≥ 60 years. Furthermore, it increased significantly to 130 mL in the patients with iNPH, whereas the mean volume of total subarachnoid space was smaller but not significantly different, compared with those in the healthy volunteers aged ≥ 60 years. All indices specific to iNPH, except callosal angle, were significantly different among the patients with iNPH and healthy volunteers aged ≥ 60 years, and among the three age groups in healthy controls.

**Fig. 3:**
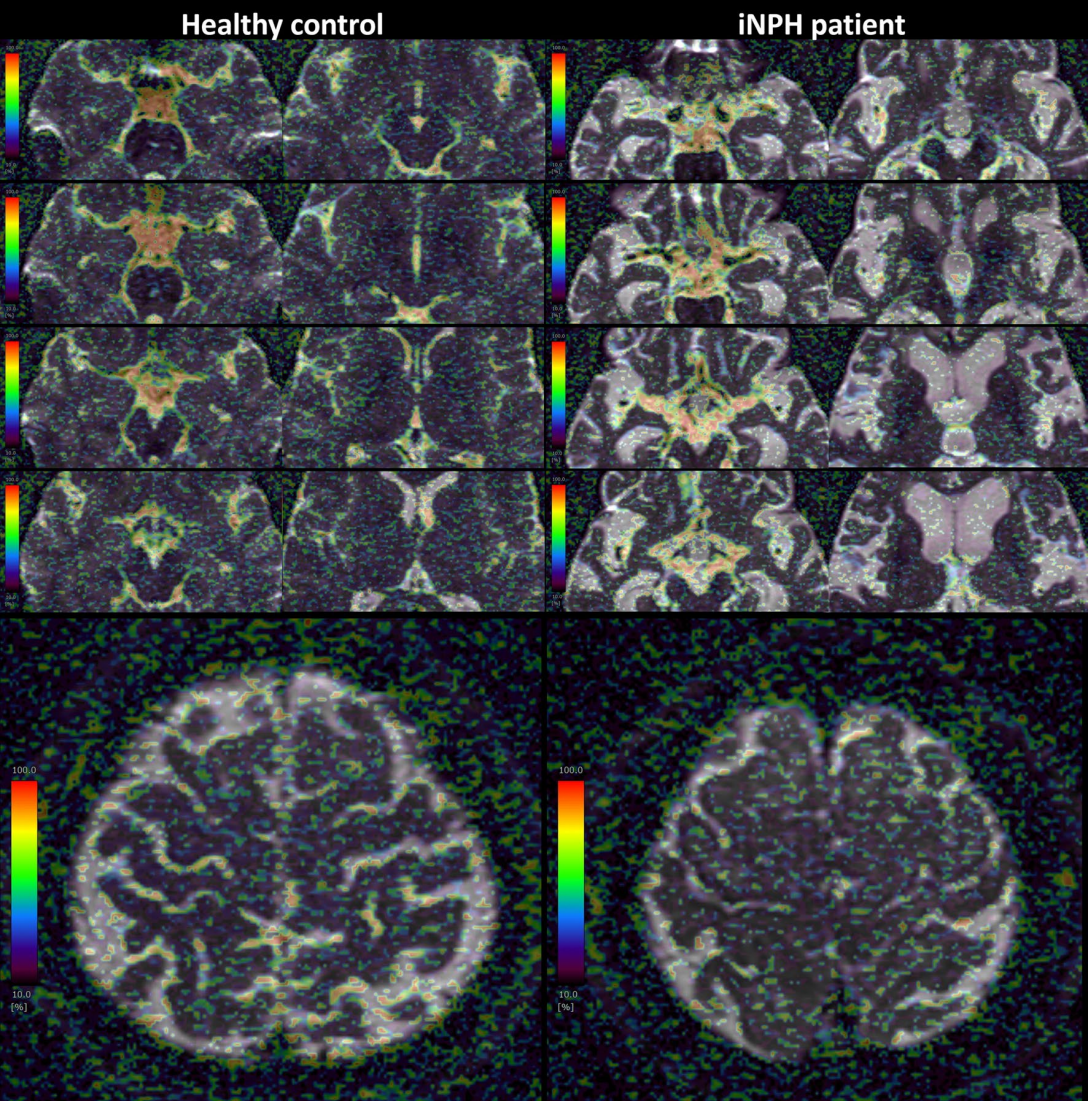
*f* map (%) of IVIM MRI in the ventricles and subarachnoid spaces in a representative healthy control (left) and iNPH patient (right).

The *f* maps calculated by IVIM analysis in the representative healthy volunteer and representative patient with iNPH are shown in Fig. 3. In the healthy volunteers, *f* values were remarkably high from the 3rd ventricle to the 4th ventricle. In addition, the healthy volunteers had high *f* values in the basal cisterns, Sylvian fossa, and prepontine cistern which contain major intracranial arteries such as the circle of Willis and basilar artery (Fig. 3). In the patients with iNPH, the enlarged ventricles and enlarged Sylvian fossae, except those close to the large arteries, did not have high *f* values. In the convexity part of the subarachnoid spaces, *f* values were high only in the sulci where relatively large arteries run, but low outside the sulci in the healthy volunteers (Fig. 3, bottom left), whereas the compressed narrow sulci at the high convexity had low *f* values in the patients with iNPH (Fig. 3, bottom right).

Compared with the healthy controls aged ≥ 60 years, the patients with iNPH had significantly lower mean *f* values in the all parts of the lateral and 3rd ventricles, but those in the cerebral aqueduct, all parts of the 4th ventricle, and foramen of Magendie were not significantly different among the patients with iNPH and healthy controls aged ≥ 60 years (Table 2). Conversely, only the bilateral foramina of Luschka showed a significantly higher mean *f* value in the iNPH group, compared with the healthy controls aged ≥ 60 years. Except for the prepontine cistern (high* f* values in both groups) and interpeduncular cistern (low *f* values in both groups), all parts of the subarachnoid spaces showed significantly lower mean *f* values in the iNPH group, compared with the healthy controls aged ≥ 60 years. Among the three age groups in healthy controls, the mean *f* values in the cerebral aqueduct, upper and middle parts of 4th ventricle, bilateral foramina of Luschka, ambient cistern, Sylvian fossa, Sylvian sulcus, and superior frontal sulcus increased significantly with increasing age (Table 2). Conversely, those in the bilateral body parts of the lateral ventricles decreased significantly with increasing age.

**Table 2:** Mean *f* values (%) of IVIM MRI in 45 regions of interests among three age group in healthy controls and patients with iNPH

Region of interest	Healthy volunteer			iNPH patient (36)	*P*-value^a^	*P*-value^b^
	< 40 years (47)	40–59 years (49)	60 < = years (36)			
Lt. lateral ventricle (anterior horn)	43.2 ± 18.6	44.0 ± 12.4	48.9 ± 19.1	23.9 ± 7.8	< 0.001	0.274
Rt. lateral ventricle (anterior horn)	43.0 ± 17.4	46.9 ± 12.0	53.8 ± 16.7	24.5 ± 8.0	< 0.001	0.017
Lt. lateral ventricle (body)	35.4 ± 14.8	33.8 ± 13.7	29.4 ± 16.5	19.9 ± 6.3	< 0.001	0.028
Rt. lateral ventricle (body)	39.2 ± 15.3	32.0 ± 11.7	27.3 ± 15.7	21.7 ± 6.2	0.108	< 0.001
Lt. lateral ventricle (trigon)	52.8 ± 15.0	53.4 ± 12.6	58.8 ± 12.2	29.4 ± 13.4	< 0.001	0.164
Rt. lateral ventricle (trigon)	53.6 ± 15.0	52.9 ± 10.0	58.6 ± 14.0	31.1 ± 11.3	< 0.001	0.112
Lt. lateral ventricle (inferior horn)	58.0 ± 13.1	56.3 ± 12.5	64.2 ± 11.5	36.9 ± 12.1	< 0.001	0.014
Rt. lateral ventricle (inferior horn)	58.1 ± 11.8	56.8 ± 11.1	66.4 ± 7.6	39.0 ± 12.2	< 0.001	< 0.001
Lt. foramen of Monro	75.1 ± 13.2	78.0 ± 10.4	79.6 ± 7.9	69.2 ± 16.9	0.005	0.419
Rt. foramen of Monro	72.0 ± 13.3	76.0 ± 10.1	78.1 ± 10.8	67.6 ± 18.2	0.008	0.041
3rd ventricle (anterior part)	81.2 ± 9.8	81.5 ± 11.2	76.1 ± 20.1	48.2 ± 25.6	< 0.001	0.755
3rd ventricle (posterior part)	82.5 ± 6.9	83.7 ± 6.8	84.6 ± 6.2	80.3 ± 8.1	0.026	0.235
Cerebral aqueduct	69.1 ± 11.1	68.0 ± 13.5	74.4 ± 9.0	74.6 ± 8.7	0.955	0.026
4th ventricle (upper part)	75.1 ± 7.2	73.1 ± 8.3	78.0 ± 5.8	75.7 ± 8.2	0.277	0.015
4th ventricle (middle part)	50.2 ± 15.9	49.7 ± 16.1	58.9 ± 17.6	54.2 ± 18.5	0.224	0.027
4th ventricle (lower part)	61.6 ± 12.1	61.3 ± 15.5	62.3 ± 14.5	67.1 ± 13.3	0.096	0.871
Lt. foramen of Luschka	49.3 ± 12.3	48.2 ± 10.5	57.8 ± 14.4	66.6 ± 14.1	0.004	0.001
Rt. foramen of Luschka	46.3 ± 8.9	46.1 ± 10.6	58.5 ± 12.6	69.0 ± 10.0	< 0.001	< 0.001
Foramen of Magendie	74.6 ± 8.4	75.3 ± 9.6	78.3 ± 7.4	78.0 ± 7.5	0.698	0.116
Foramen magnum	75.7 ± 12.3	69.0 ± 14.4	77.7 ± 11.8	62.7 ± 20.7	< 0.001	0.008
Lt. cerebellopontine angle	84.1 ± 8.2	85.5 ± 6.4	85.9 ± 6.9	80.3 ± 10.8	0.023	0.523
Rt. cerebellopontine angle	83.8 ± 8.2	86.5 ± 7.0	88.0 ± 5.6	79.2 ± 11.8	< 0.001	0.022
Prepontine cistern	88.5 ± 3.9	87.6 ± 3.5	88.4 ± 3.2	87.1 ± 6.1	0.719	0.359
Suprasellar cistern	89.7 ± 4.7	90.2 ± 2.6	89.9 ± 3.7	83.9 ± 12.6	0.006	0.952
Interhemispheric fissure (lower part)	80.9 ± 8.1	82.8 ± 7.0	85.3 ± 6.3	78.9 ± 13.1	0.011	0.033
Quadrigeminal cistern	68.0 ± 11.9	73.0 ± 9.4	71.4 ± 15.5	60.2 ± 16.4	0.001	0.081
Lt. ambient cistern	82.4 ± 9.5	84.9 ± 4.2	86.8 ± 5.4	71.5 ± 15.3	< 0.001	0.013
Rt. ambient cistern	83.2 ± 8.7	84.5 ± 5.6	88.6 ± 3.9	73.0 ± 15.0	< 0.001	< 0.001
Lt. carotid cistern	89.3 ± 4.2	89.3 ± 4.8	91.9 ± 1.9	86.1 ± 7.7	< 0.001	< 0.001
Rt. carotid cistern	89.9 ± 4.4	89.7 ± 3.5	91.3 ± 2.0	86.7 ± 6.2	< 0.001	0.097
Lt. Sylvian fossa	75.1 ± 16.6	80.9 ± 12.1	89.4 ± 4.6	62.6 ± 22.8	< 0.001	< 0.001
Rt. Sylvian fossa	72.8 ± 17.4	80.2 ± 11.0	89.1 ± 5.4	63.2 ± 22.2	< 0.001	< 0.001
Lt. Sylvian sulcus (anterior ramus)	56.0 ± 14.8	60.7 ± 12.0	69.9 ± 10.6	49.4 ± 18.9	< 0.001	< 0.001
Rt. Sylvian sulcus (anterior ramus)	56.3 ± 14.7	65.3 ± 10.7	69.6 ± 9.1	51.6 ± 18.7	< 0.001	< 0.001
Lt. Sylvian sulcus (posterior ramus)	69.0 ± 14.2	73.9 ± 9.6	81.3 ± 5.6	56.8 ± 20.1	< 0.001	< 0.001
Rt. Sylvian sulcus (posterior ramus)	69.1 ± 12.9	75.2 ± 9.9	80.8 ± 6.4	57.1 ± 18.4	< 0.001	< 0.001
Lt. central sulcus	52.4 ± 13.4	54.6 ± 10.0	58.8 ± 11.0	36.9 ± 10.4	< 0.001	0.035
Rt. central sulcus	53.7 ± 12.6	53.8 ± 9.5	56.9 ± 14.0	39.9 ± 10.9	< 0.001	0.347
Lt. superior frontal sulcus	45.6 ± 13.7	49.5 ± 10.7	55.2 ± 14.1	36.6 ± 12.3	< 0.001	0.008
Rt. superior frontal sulcus	46.1 ± 15.2	51.4 ± 10.8	57.3 ± 12.0	36.6 ± 11.1	< 0.001	< 0.001
Lt. marginal sulcus	55.8 ± 12.0	59.9 ± 11.5	59.0 ± 13.5	38.7 ± 12.4	< 0.001	0.140
Rt. marginal sulcus	56.3 ± 12.4	59.4 ± 11.1	57.0 ± 14.9	36.2 ± 12.4	< 0.001	0.413
Interhemispheric fissure (upper part)	41.5 ± 17.0	48.1 ± 16.6	54.0 ± 16.7	35.2 ± 16.9	< 0.001	0.011
Lamina terminals cistern	47.6 ± 18.5	39.2 ± 16.2	37.7 ± 18.3	29.3 ± 10.4	0.033	0.041
Interpeduncular cistern	39.0 ± 11.4	34.5 ± 12.6	35.0 ± 15.3	30.9 ± 13.3	0.290	0.066

^a^*P*-value: probability value for the Mann–Whitney-Wilcoxon test between iNPH patients and healthy volunteer aged ≥ 60 years

^b^*P*-value: probability value for the Kruskal–Wallis test among 3 age groups of healthy volunteer

**Fig. 4:**
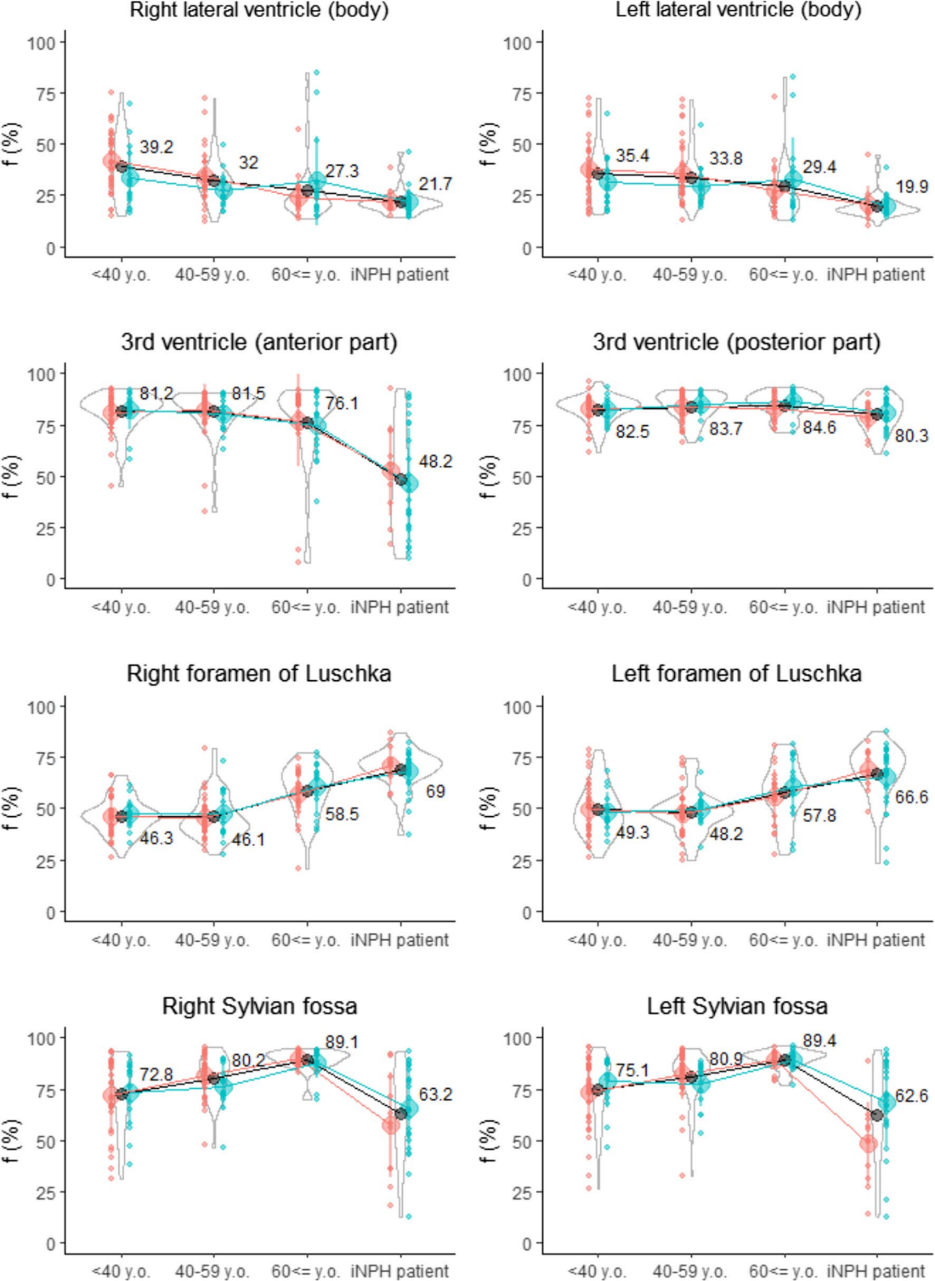
Distribution of *f* value (%) on IVIM MRI among healthy controls and iNPH patients. Red indicates female, blue indicates male, and black indicates total. The center circle and vertical lines denote the median and the 25% and 75% interquartile ranges for each group. The violin plots show the distribution for the *f* value in the total subjects. The numbers in the graph are the mean f value (%) in each group

As shown in Fig. 4, the mean f values in the bilateral body parts of the lateral ventricles decreased gradually with increasing age, and further lower in the iNPH patients. In the anterior part of the 3rd ventricle, the mean f value began to decline from the healthy controls aged ≥ 60 years, and dropped sharply in the iNPH patients, although that in the posterior part of the 3^rd^ ventricle maintained high in the all groups. Conversely, those in the bilateral foramina of Luschka began to increase from the healthy controls aged ≥ 60 years, and further higher in the iNPH patients. In the bilateral Sylvian fossa, which contain the middle cerebral bifurcation, the mean f values increased gradually with increasing age, whereas those were significantly lower in the iNPH patients (Fig. 4 bottom).

In the 45 ROIs, the mean f value in the anterior part of the 3rd ventricle was the most negatively correlated with the ventricular volume (Pearson’s correlation coefficient: − 0.62), Z-Evans index (− 0.56), and the most positively correlated with the BVR at AC (0.54), and callosal angle (0.53). Conversely, the mean *f* values in the right and left foramina of Luschka was positively correlated with the ventricular volume (0.56 and 0.45), total volume of the subarachnoid spaces (0.43 and 0.34), Evans index (0.30 and 0.32), Z-Evans index (0.62 and 0.48), and negatively correlated with the BVR at AC (-0.62 and -0.47), BVR at PC (-0.60 and -0.47), and callosal angle (-0.49 and -0.42).

Other parameters of ADC, D, and D* were not significantly different between any two groups in any locations.

## Discussion

IVIM MRI with low multi-b DWI could visualize and quantify the small pulsatile complex CSF motion that could not be evaluated by conventional flow-related MRI. Because IVIM MRI does not require synchronization of heartbeat or respiration, it has the potential to provide an overview of all CSF movements driven by arterial pulsations, brain pulsations produced by the cerebral blood circulation, respiration, and coordinated directional beating of the motile cilia, other than head movements. In addition, the mean *f* value calculated by IVIM MRI is useful as a quantitative value of incoherent motion of the CSF. The fact that the IVIM parameter, *f*, measures CSF movements has been reported,[19, 23, 36] but to our knowledge there have been no reports of *f* values being measured both in the whole ventricles and subarachnoid spaces. The mean *f* values in all parts of the lateral ventricles and anterior part of the 3rd ventricle were significantly decreased in the iNPH group compared with the healthy group. These findings suggest that the CSF around the ventricular walls of the lateral ventricle flows rarely in iNPH because of a reduction in brain pulsations and a weakness or dysfunction of ciliary movements of ependymal cells on the ventricle surfaces [1, 2, 7]. The mean *f* value in the bilateral foramina of Luschka was significantly higher in the iNPH group than in the healthy group, although the values in the foramen of Magendie were high in both groups. Previous studies on CSF motions measured by conventional phase-contrast MRI and four-dimensional flow MRI have shown a significant increase in CSF stroke volumes at the cerebral aqueduct, foramen of Magendie, and foramen magnum in the iNPH group compared with the healthy group [3, 5–7, 12, 18]. However, no study has mentioned the CSF motion in the bilateral foramina of Luschka. The foramen of Magendie and bilateral foramina of Luschka are well-known pathways connecting the 4th ventricle and the subarachnoid space in the posterior fossa, but their stroke volumes had not been compared. Our finding suggests that the foramen of Magendie is the main pathway, and the bilateral foramina of Luschka are the secondary pathways through which CSF does not always pass through and may be a temporary route only when CSF levels increased.

Compared with the healthy aging brain, the iNPH had a small amount of the CSF that barely flows in the central sulcus and marginal sulcus because of compression by the simultaneous expansion of the ventricles and Sylvian fissure toward the top of the head [7, 30, 33, 34, 37]. These findings indicates a dysfunction of the glymphatic system in which CSF flows along the periarterial space surrounding the penetrating cerebral arteries from the sulci at the convexity part of subarachnoid space in patients with iNPH [13–17]. Furthermore, the *f* values in the Sylvian fissure, which contains the middle cerebral arteries increased gradually with increasing age, whereas those were significantly lower in the iNPH group. The increase in pulsatile CSF motion in the Sylvian fissure with aging would be associated with the increased pulsatile blood flow in cerebral arteries due to aortic stiffness [38], and the decreased pulsatile CSF motion in iNPH would be associated with the enlargement of the Sylvian fissure. Although the small pulsatile complex motion of the CSF and interstitial fluid is thought to be mainly driven by arterial pulsations in the glymphatic system [13, 14], Taoka et al. reported that the driving force of CSF pulsation in the Sylvian fissure may be related to the pulsations of the cerebral hemisphere rather than direct arterial pulsations in the study of the unilateral middle cerebral artery occlusion on DWI with b-value of 500 s/mm^2^ [22].

IVIM MRI has several advantages compared with the four-dimensional flow MRI or time-SLIP MRI. The most important advantage is a short acquisition time of approximately 2 min in this study. The quantitativeness of CSF motion in a wide range of flow velocity and wide area including the whole ventricles and subarachnoid spaces is also an important advantage. Therefore, IVIM MRI is a potentially useful and convenient technique for the evaluation of CSF dynamics. The disadvantages of IVIM MRI are its low resolution and inability to measure the flow direction of the CSF movements.

This study has some other limitations. First, the distributions of gender and age in the healthy controls aged ≥ 60 years did not match those in the iNPH group. The mean age in the iNPH group was significantly higher than that in the healthy controls aged ≥ 60 years group. A sufficient number of older controls could have shown more clearly the difference in *f* value between iNPH and healthy aging brain. Second, how much the *f* value of the CSF reflects the flow velocity was not evaluated. Le Bihan et al. reported that the *f* value of water decreases as its flow velocity decreases, but no linear relationship was observed in the phantom study [23]. Third, the bi-exponential IVIM fitting method was chosen for the IVIM analysis, but the complex CSF motions may contain numerous components. Fourth, the ROIs were manually placed by a single researcher based on anatomical features, so reproducibility was not ensured. We would like to enable automatic ROI placement in the application. Finally, the slice thickness of DWI was set at 3 mm in this study. Thick slice spacing and low resolution may reduce the reliability of the *f* value on IVIM MRI, especially in a small lumen structure such as the cerebral aqueduct.

## Conclusions

IVIM MRI could visualize and quantify the small pulsatile complex motion of the CSF in the whole intracranial CSF spaces, including the central sulcus. The mean *f* value on IVIM MRI is a potential index useful for evaluating complex CSF motion, although their direction and flow velocity could not be examined. Quantitative measurement of small CSF motions in the whole intracranial CSF spaces may contribute to the elucidation of the CSF dynamics and glymphatic function in iNPH and aging effects. We hope to establish a simulation model of the intracranial biological and metabolic environment by integrating* f* values on IVIM MRI and 3D-flow velocities on 4D Flow MRI in addition to 3D morphology information.

## Data Availability

The MRI data in this study is not available to the community via any open repositories, because the ethics committees have approved that this research shares MRI data with five collaborative institutes and does not allow it to be provided to other institutions. The data will only be available if the ethics committees approve new participation in the collaborative research.

## Abbreviations

ADC: Apparent diffusion coefficient
BVR: Brain per ventricle ratio
CSF: Cerebrospinal fluid
D: Diffusion coefficient
D*: Pseudo-diffusion coefficient
DESH: Disproportionately enlarged subarachnoid space hydrocephalus
DWI: Diffusion weighted image
f: Fraction of incoherent perfusion
IVIM: Intravoxel incoherent motion
iNPH: Idiopathic normal pressure hydrocephalus
PC: Phase-contrast
ROI: Region of interest
SD: Standard deviation
SLIP: Spatial labeling inversion pulse
